# Reframing Anesthetic Principles: Telesurgery as the Natural Evolution of Robotic Surgery

**DOI:** 10.1590/S1677-5538.IBJU.2025.0247

**Published:** 2025-05-30

**Authors:** Giovana Barani, Marcio Covas Moschovas, Belmira Luís, Bruno Gallo, Vipul Patel

**Affiliations:** 1 AdventHealth Global Robotics Institute Kissimmee FL USA AdventHealth Global Robotics Institute, Kissimmee, FL, USA; 2 University of Central Florida FL USA University of Central Florida - UCF, FL, USA; 3 Complexo Hospitalar Cardeal Dom Alexandre do Nascimento Luanda Angola Complexo Hospitalar Cardeal Dom Alexandre do Nascimento, Luanda, Angola

## COMMENT

We read with great interest the article by Wang and colleagues describing the anesthesia perspective in telesurgery procedures (1). The expansion of telesurgery through the integration of high-speed 5G networks has enabled the remote delivery of surgical care using robotic systems (2, 3). This development has been highlighted for its potential to democratize access to high-quality surgical expertise, particularly in regions with limited local resources (4, 5). However, the anesthetic management of these procedures is often portrayed as novel or fundamentally different. We argue that telesurgery is an extension of robotic surgery; thus, the anesthetic principles applied to robotic procedures should also govern telesurgical practice (6–8).

In this context, considering that telesurgery is a form of robotic surgery, the foundational anesthetic principles—general anesthesia with deep muscle relaxation, appropriate monitoring, and multimodal analgesia—apply equally to telesurgery (6). The patient's physiology and the type of procedure are not affected by the geographic location of the surgeon. Consequently, from the anesthesiologist's perspective, anesthetic goals remain unchanged: to ensure immobility, hemodynamic stability, adequate ventilation, and rapid recovery. Specific considerations such as patient positioning, pneumoperitoneum effects, neuromuscular blockade, and temperature regulation are identical to traditional robotic cases and should be managed accordingly. Furthermore, robotic surgery technology has been established for several years, and even the most recent platforms have demonstrated safety in clinical settings before market release (9, 10). Therefore, the robotic platform—whether operated locally or remotely—offers the same performance and safety for patients, and communication between anesthesia providers and local surgeons remains unchanged.

On-site surgical expertise is imperative to ensure patient safety and optimal outcomes (11, 12). Despite the advanced capabilities of remote control in telesurgical procedures, the physical presence of an experienced surgeon in the operating room is non-negotiable (8). This individual plays a critical role in managing potential intraoperative complications, such as unexpected bleeding, conversion to open surgery, or system failure due to signal loss. Their immediate availability ensures procedural continuity and safeguards patient safety during high-stakes or time-sensitive events.

From the anesthetic standpoint, coordination with the local surgeon is equally essential, especially when sudden changes in the surgical plan require prompt anesthetic adjustments. However, it is important to emphasize that anesthetic management in telesurgery remains fundamentally the same as in conventional robotic surgery. Regardless of the scenario, the anesthesiologist prepares and monitors the patient as they would for any standard robotic procedure. This consistency is made possible by the presence of the local surgical team, who can intervene directly and promptly if needed. As a result, the anesthesia team can rely on established protocols, ensuring safety and stability throughout the case while maintaining seamless communication with both local and remote surgical teams.

Another important aspect discussed in the article is the potential impact of remote surgeon performance on anesthesia management and patient recovery. It is essential to highlight that when an expert telesurgeon is in control, the benefits extend well beyond surgical precision (8). Highly experienced telesurgeons can simplify complex operations by minimizing unnecessary instrument movements, avoiding indecision, and executing each step efficiently. This results in shorter operative times, directly reducing anesthetic exposure.

From an anesthetic perspective, this reduction is highly beneficial. Shorter anesthesia durations are associated with decreased risks of intraoperative hypothermia, lower incidence of postoperative delirium—particularly in elderly or vulnerable patients—and faster recovery, all contributing to a smoother and more predictable postoperative course. Additionally, shorter procedures reduce the need for prolonged intraoperative support measures such as fluid resuscitation or vasopressor use, improving overall patient stability. These clinical benefits also carry important economic implications. Reduced operative time leads to more efficient use of operating room resources, decreased staffing demands, and lower overall procedural costs (13). Patients benefit not only from a safer anesthetic experience but also from reduced hospital stays, earlier mobilization, and a quicker return to daily activities. In this context, the involvement of a highly skilled telesurgeon generates a cascade of anesthetic and systemic advantages, improving patient outcomes while reducing the healthcare burden.

The authors also emphasize the importance of communication between the remote surgeon and the anesthesia team. While this is a valid point, it is important to clarify that such communication is already an inherent and well-established component of any telesurgical setup. As with conventional robotic procedures, there is continuous and structured communication between the console surgeon, the bedside assistant, and the anesthesia providers. The local surgeon, physically present with the patient, remains fully informed of the intraoperative course and serves as a key intermediary, relaying any necessary updates to the remote surgeon in real time. Modern telesurgical systems utilize stable, secure communication channels that function seamlessly throughout the procedure, typically through encrypted platforms or direct audio connections via smartphone technology. In this regard, communication in telesurgery closely mirrors current practices in many operating rooms, where surgical teams use headsets, video monitors, and team-based communication protocols.

Thus, the notion that communication presents a significant barrier in telesurgery is largely overstated. The setup is intuitive and comparable to established workflows for both the anesthesia team and the remote surgeon. Even in high-pressure situations such as trauma or intraoperative emergencies, the presence of a local surgeon mitigates delays in decision-making or intervention, ensuring that patient safety is not compromised. Effective communication in telesurgery is not a limitation but a built-in feature that enables fluid collaboration across distances with the same reliability as traditional robotic procedures.

Moreover, when establishing a telesurgery program, simulation and training for integrated teams are essential. Given the unique configuration of telesurgical teams, we recommend incorporating simulation training that includes anesthesiologists, local surgical staff, and remote surgeons. Scenarios should address network latency, device failure, and intraoperative emergencies to ensure that all team members can respond efficiently and cohesively. These simulations should also evaluate nontechnical skills such as communication clarity, teamwork, and leadership in distributed environments.

Another point raised by the authors concerns the potential health risks of "5G radiation exposure" during telesurgery—a claim that lacks scientific foundation and may be misleading. Although isolated studies have explored the biological effects of 5G, there is currently no conclusive evidence linking standard 5G exposure to harmful health outcomes. Importantly, 5G technology is already integrated into daily life through smartphones, wireless devices, and urban infrastructure, often for extended periods (2). In the context of telesurgery, 5G is used exclusively for data transmission between surgical sites and does not involve direct or prolonged exposure to the patient or surgical team. The intensity and duration of 5G exposure in these procedures are minimal and comparable to everyday mobile technology use. While continued research into the long-term effects of emerging technologies is appropriate, highlighting 5G in telesurgery as a unique health risk is not scientifically justified and detracts from the broader benefits and established safety of these communication systems.

Finally, ethical considerations for anesthesiologists in telesurgery should include aspects of informed consent. Patients must be appropriately informed about the nature of telesurgical procedures, including the potential risks associated with remote operation and network dependency (8). Although surgeons typically lead these discussions, anesthesia providers are responsible for clearly communicating perioperative anesthetic risks within this novel context. Anesthesiologists participating in 5G-enabled robotic surgery face distinct challenges, including patient immobility, real-time monitoring, remote coordination, network reliability, emergency management, pain control, and the need for specialized training. By following current recommendations—such as employing general anesthesia with deep muscle relaxation, enhancing monitoring, ensuring robust communication, preparing for network disruptions, planning for emergencies, implementing multimodal analgesia, and participating in simulation training—anesthesiologists can provide safe and effective care.

As telesurgery becomes more widespread, the role of the anesthesiologist will grow in both complexity and importance. However, we must resist the urge to reinvent foundational principles. Telesurgery is a natural evolution of robotic surgery, in which procedures are performed remotely. By applying established anesthetic protocols, ensuring the presence of on-site surgical support, and investing in structured communication strategies, we can safely incorporate telesurgery into routine clinical practice. Prioritizing these principles will enhance procedural safety, improve patient outcomes, and support the expansion of surgical access across geographic boundaries.
